# Unravelling the genetics of non-random fertilization associated with gametic incompatibility

**DOI:** 10.1038/s41598-022-26910-8

**Published:** 2022-12-24

**Authors:** Audrey A. A. Martin, Samir Id-Lahoucine, Pablo A. S. Fonseca, Christina M. Rochus, Lucas M. Alcantara, Dan Tulpan, Stephen J. LeBlanc, Filippo Miglior, Joaquim Casellas, Angela Cánovas, Christine F. Baes, Flavio S. Schenkel

**Affiliations:** 1 Centre for Genetic Improvement of Livestock, University of Guelph, Guelph, ON Canada; 2 Department of Animal and Veterinary Sciences, Scotland’s Rural College, Edinburgh, Scotland, UK; 3 Ontario Veterinary College, University of Guelph, Guelph, ON Canada; 4 Departament de Ciència Animal I Dels Aliments, Universitat Autònoma de Barcelona, Bellaterra, Spain; 5 Institute of Genetics, Vetsuisse Faculty, University of Bern, Bern, Switzerland

**Keywords:** Agricultural genetics, Animal breeding, Functional genomics, Genetics, Haplotypes, Reproductive biology

## Abstract

In the dairy industry, mate allocation is dependent on the producer’s breeding goals and the parents’ breeding values. The probability of pregnancy differs among sire-dam combinations, and the compatibility of a pair may vary due to the combination of gametic haplotypes. Under the hypothesis that incomplete incompatibility would reduce the odds of fertilization, and complete incompatibility would lead to a non-fertilizing or lethal combination, deviation from Mendelian inheritance expectations would be observed for incompatible pairs. By adding an interaction to a transmission ratio distortion (TRD) model, which detects departure from the Mendelian expectations, genomic regions linked to gametic incompatibility can be identified. This study aimed to determine the genetic background of gametic incompatibility in Holstein cattle. A total of 283,817 genotyped Holstein trios were used in a TRD analysis, resulting in 422 significant regions, which contained 2075 positional genes further investigated for network, overrepresentation, and guilt-by-association analyses. The identified biological pathways were associated with immunology and cellular communication and a total of 16 functional candidate genes were identified. Further investigation of gametic incompatibility will provide opportunities to improve mate allocation for the dairy cattle industry.

## Introduction

Reproduction is a main concern for the dairy industry, as lactation is dependent on the capacity of a cow to produce a calf. Moreover, the fertility of high genetic merit animals is of utmost importance, as these animals are the parents of the next generation. Nevertheless, while female fertility has been part of national selection indexes for dairy cattle around the world over the past decades, the same is not observed for male fertility^1,2^. Moreover, there is a low genetic correlation between male and female fertility traits, which along with the low heritability of these traits, indicate that the indirect response to selection on female fertility traits would not be sufficient to improve male fertility^1^. The males’ contribution needs to be considered for the general improvement of reproductive capacity, as it is an integral component of fertilization. For example, genomic selection for sire conception rate is feasible but complicated^3^. The true reproduction ability and natural variation of bull’s conception rate have been masked by the necessary standardization of semen for the widespread use of artificial insemination (AI). Semen doses are extended and titrated to reach an acceptable probability of pregnancy per AI across the population based on sperm characteristics for young bulls and later adjusted when sire conception rate phenotypes are available, which reduces the observed variation in male fertility^1^.

Fertilization success is strongly dependent on the compatibility of gametes. The paired “lock and key” mechanism of the spermatozoa and the oocyte is essential and relies on the proper interaction between the proteins of both gametes, which is also called gametic compatibility^4,5^. It is most studied in externally fertilizing species, such as fish and sea urchin, where the strong interspecies gametic incompatibility replaces the physical isolating barrier^4^. More recently, the biological patterns of gametic interaction have been investigated in mammals, e.g. gamete-mediated mate choice^6^ and cryptic female choice^7^.

Under Mendel’s first law, alleles from the same locus should segregate independently, so fertilization, the union of two gametes, should create an equal genotypic ratio in the offspring generation. However, exceptions due to different mechanisms are known, resulting in a biased ratio of genotypes within the descendants^8^. This bias can be investigated with a transmission ratio distortion (TRD) analysis, which identifies if one of the two parental alleles is over- or under-represented in the descendants. Transmission ratio distortion is therefore defined by a deviation from the Mendelian law of inheritance and is linked to diverse mechanisms, from gametic formation to embryo development^9^. A few recent studies used TRD to investigate gametic compatibility (e.g. in mice^10^ and plants^11^), but to our knowledge, no studies have explored genetic incompatibility in livestock species. To study possible gametic incompatibility, the TRD model from Casellas et al.^12,13^ was adapted by including a gametic interaction term in addition to the direct TRD effect, which is based on the parental allele transmission differential.

The complete or partial genetic incompatibility of gametes produces an unbalanced genotypic frequency in the offspring population. This study aimed to uncover genomic regions and to identify candidate genes associated with incompatible gametic combinations, as well as to apply network, over-representation, and guilt-by-association analyses to discover the underlying pathways of gametic incompatibility. In the long-term, and with further investigation, mate allocation could potentially be improved by avoiding mating that are incompatible or have a lesser chance of successful pregnancy.

## Results

### TRD regions and positional genes

After running the TRD model with the additional gametic interaction term, a total of 482 genomic regions (5217 allelic combinations) were identified as having one or both significant direct and gametic interaction TRD effects. After applying filtering for strong gametic interaction, 429 unique regions were left and 422 were successfully remapped to the ARS-UCD1.2 assembly. The complete list of genomic regions with the corresponding positional genes is reported in Supplementary Material Table S1 with the chromosomal position of each region and the Ensembl ID of every gene contained in each region. All the supplementary materials (Tables and Figures) are available at https://aaamartin.shinyapps.io/netview/. The genomic regions contained 2075 Ensembl gene IDs, including 490 without annotation. The number of positional candidate genes within each *Bos taurus* autosome (BTA) is presented in supplementary material Table S2. Compared to the total gene distribution, there were more genes associated with gametic interaction on BTA 10, and fewer on BTA 18.

### Network analysis

Creating a minimum network, i.e. reducing the network to a minimum of interactions by only keeping the shortest pair-wise paths between genes with NetworkAnalyst^14^ (www.networkanalyst.ca) resulted in 13 subnetworks, one continent (399 nodes) and 12 islands (3 to 11 nodes each). A supplementary network was created when a positional gene could not be connected with an already existing network. Only the first subnetwork had a substantial number of genes and related pathways, therefore only this network was further reported and discussed. However, the complete results, including each network and related Kyoto Encyclopedia of Genes and Genomes (KEGG) pathways^15,16^ and Gene Ontology (GO) terms are in the supplementary materials Fig. S1 and Table S3 (https://aaamartin.shinyapps.io/netview/), respectively.

There were 120 significantly enriched KEGG pathways (false discovery rate; FDR < 0.05) in the network. Most pathways were related to immunological and signalling functions: 44 KEGG pathways directly related to disease (due to perturbations of biological networks), e.g. “Hepatitis B” and “Pancreatic cancer”; 14 to different actors or processes of the immune system, such as “Th17 cell differentiation” and “Leukocyte transendothelial migration”; and 40 related to diverse signalling processes, e.g. “Rap1 signalling pathway” and “Thyroid hormone signalling pathway”. There were a few pathways associated with reproduction mechanisms including: “GnRH signalling pathway”, “Prolactin signalling pathway”, “Progesterone-mediated oocyte maturation”, “Oocyte meiosis”, “Oestrogen signalling pathway”, and the “Oxytocin signalling pathway”. The other significant KEGG terms usually referred to central cellular mechanisms, for example the MAPK (mitogen-activated protein kinases) signalling pathway that participates in the regulation of cell proliferation, differentiation, motility, and survival^17^.

For the GO terms analysis of the network, 55 GO-BP, 51 GO-MF and 38 GO-CC were enriched. Like the KEGG pathway analysis, terms related to immunologic functions, such as “Inflammatory response” and “Positive regulation of cytokine secretion”, were present. Additionally, terms relating to different mechanisms associated with the regulation of genetic materials, such as “DNA replication initiation” (GO-BP), “Nucleotide binding” (GO-MF) and “Chromosome” (GO-CC), were abundant.

### Overrepresentation analysis

Similar to the network analysis results, the overrepresentation analysis (ORA) pointed to communication, immunological, and metabolic functions but not directly to reproduction processes. It can be noted that only the analysis using GO-CC terms, presented in Table 1, resulted in significant enrichment (FDR < 0.05). Most terms referred to entities carrying or processing the genetic material within the cell, such as chromosome and endoplasmic reticulum. Based on the enrichment ratio, processes involving the aggresome were the most overrepresented. An aggresome is an inclusion body that can transport aggregated proteins on the cytoskeleton to have them “recycled”. It is linked to the autophagy process where the cell recycles one of its components, which is also found in the network analysis (Networks 1 and 12). This process is involved in cellular structural changes.

**Table 1:** Significantly overrepresented GO-CC terms for a list of 2075 Ensembl gene ID positioned in transmission ratio distortion genomic regions associated with strong gametic interaction.

GO-CC	Total	Expected	Hits	Ratio	FDR
Aggresome	18	1	6	5.71	0.03
Nuclear chromosome	302	18	34	1.93	0.03
Nuclear chromosome part	287	17	34	1.91	0.03
Chromosomal part	525	31	52	1.70	0.03
Chromosome	573	33	55	1.64	0.03
Ribonucleoprotein complex	573	33	54	1.61	0.03
Endoplasmic reticulum	875	51	76	1.49	0.03
Nucleoplasm	1444	84	120	1.42	0.01
Cytosol	1795	105	146	1.39	0.01
Nuclear lumen	1937	113	148	1.31	0.03

*GO-CC* Gene Ontology-Cellular Component, *Total* total number of genes in the genome associated with each GO term, *Expected* expected number of genes within the network dataset associated with each GO term, *Hits* observed number of genes within the network dataset associated with each GO term, *Ratio* Ratio of overrepresentation, *FDR* false discovery rate.

Table 2 shows the ten most enriched KEGG pathways, in which most of them were related to immunology and diseases. Specifically, the process of self and non-self recognition appears to be important, because the highest ratios of overrepresentation were associated with pathways relating to autoimmune diseases, for which the recognition process is defective. However, these results were not statistically significant at a FDR of 5%, therefore they should not be used on their own, but rather as an additional information for the interpretation of the significant results from other analyses in combination with the available literature.”

**Table 2:** First 10 most overrepresented KEGG pathways for a list of 2075 Ensembl gene ID from genes positioned in transmission ratio distortion genomic regions associated with strong gametic interaction.

KEGG Pathway	Total	Expected	Hits	Ratio	FDR
Glycosphingolipid biosynthesis	16	1	5	5.37	0.24
Graft-versus-host disease	47	3	8	2.92	0.26
Type I diabetes mellitus	58	3	9	2.66	0.26
Th17 cell differentiation	112	7	14	2.15	0.26
Gastric cancer	153	9	17	1.91	0.26
Endocytosis	246	14	26	1.81	0.24
Systemic lupus erythematosus	181	11	19	1.80	0.26
Human T-cell leukemia virus 1 infection	268	16	27	1.73	0.26
Human papillomavirus infection	354	21	32	1.55	0.26
Metabolic pathways	1329	77	104	1.34	0.20

*KEGG* Kyoto Encyclopedia of Genes and Genomes, *Total* total number of genes associated with the KEGG pathway, *Expected* expected number of genes within the network dataset associated with each KEGG pathway, *Hits* observed number of genes within the network dataset associated with each KEGG pathway, *Ratio* Ratio of overrepresentation, *FDR* false discovery rate.

### Guilt-by-association analysis

The identification of the most likely functional candidate genes with the guilt-by-association analysis resulted in 12 genes associated with fertilization, gametic interaction, and recognition. The genes are presented in Table 3. An important note was the presence of *CD9*, which is involved in the fusion of gametes, and was already proposed as a candidate gene for gametic incompatibility^4^. From the gene prioritization based on the functional profile similarity with the 12 candidate genes mentioned above, there were four significant genes (FDR < 0.05), which are presented in Table 4.

**Table 3:** List of candidate genes related to gametic incompatibility based on the keywords “fertilization”, “gamete interaction”, “single fertilization” and “sperm-egg recognition”, present in a list of 2075 Ensembl gene ID from genes positioned in transmission ratio distortion genomic regions associated with strong gametic interaction.

Gene Name	BTA	Start (bp)	End (bp)	Description
*CATSPERD*	7	18,552,688	18,590,398	Cation channel sperm associated auxiliary subunit delta
*CD9*	5	104,123,604	104,159,285	CD9 molecule
*DMC1*	5	110,195,281	110,232,976	DNA meiotic recombinase 1
*GNPDA1*	7	53,066,435	53,077,719	Glucosamine-6-phosphate deaminase 1
*MAEL*	3	1,829,695	1,901,654	Maelstrom spermatogenic transposon silencer
*MCM9*	9	32,100,137	32,225,167	Mini-chromosome maintenance 9 homologous recombination repair factor
*MFGE8*	21	20,467,445	20,503,982	Milk fat globule EGF and factor V/VIII domain containing
*PCSK4*	7	43,888,435	43,897,837	Proprotein convertase subtilisin/kexin type 4
*PRND*	13	47,086,007	47,091,045	Prion like protein doppel
*SLC9B1*	6	21,861,338	21,928,497	Solute carrier family 9 member B1
*SMAD4*	24	50,524,995	50,577,277	SMAD family member 4
*UBE2Q1*	3	16,022,438	16,032,162	Ubiquitin conjugating enzyme E2 Q1

*BTA* Bos taurus autosome, *bp* base pair.

**Table 4:** Prioritized candidate genes relating to gametic incompatibility identified by guilt-by-association analysis in a list of 2075 Ensembl gene ID from genes positioned in transmission ratio distortion genomic regions associated with strong gametic interaction.

Gene Name	BTA	Start (bp)	End (bp)	Description	FDR
ANXA1	8	49,321,701	49,339,582	Annexin A1	0.01
IFNG	5	45,624,513	45,629,336	Interferon gamma	0.01
MET	4	51,620,793	51,735,647	MET proto-oncogene, receptor tyrosine kinase	0.02
ATP2B4	16	1,398,176	1,500,871	ATPase plasma membrane Ca2 + transporting 4	0.03

*BTA* Bos taurus autosome, *bp* base pair, *FDR* false discovery rate.

## Discussion

Little is known about gametic incompatibility in mammals and few associated genes and mechanisms have been identified in the last decades^4^. The functional analyses in this study revealed that genomic regions associated with gametic interaction TRD and gametic incompatibility are mostly linked to immunology and communication pathways. It was expected because reproduction and immunology are often intertwined, relying both on cellular communication. Modulation of immunity in the uterus is necessary to protect against pathogens yet allow the survival of allogeneic cells, such as spermatozoa and foetal tissue^18^. Immunology and fertility also share a similar genetic architecture. They are both complex traits, meaning that they result from the expression of numerous genes with small effects. These traits are influenced by the environment and are strongly affected by natural selection pressure. The genetic structure of complex traits allows for the maintenance of mechanisms that are central to animal fitness, while permitting important polymorphisms necessary for adaptation. The main function of the traits remains unchanged, but it is regulated by a tight gene network, which allows for a lot of potential variation^19^.

Interestingly, purely reproductive mechanisms seemed not to be of primary importance for gametic compatibility variation. Some sexual hormone pathways and oocyte development processes were significant in this study, but they are more likely to affect fertilization by producing a sub-optimal environment for the gametes or the embryo. Likewise, the significance of many architectural processes necessary for the general function of the organism was more closely linked to the viability of the embryo than the combination of the gametes. Attributing TRD to specific biological mechanisms is complicated. First, the effects of a disease or phenotypic preselection of the studied animals could create a false TRD signal and confound the interpretation^9^. Preselection bias was mostly avoided with the chosen method for collecting genotypes (described in Materials and Methods, Dataset). Therefore, observed TRD could be attributed to diverse biological mechanisms such as germline selection, meiotic drive, gametic competition, imprinting errors, and embryo lethality^9^. By considering the interaction between the gametic haplotypes, focus was given to mechanisms taking effect in a diploid cell, i.e. at or after fertilization. This specific bias is usually attributed to lethal mutations or lower fitness genotypes in the embryo or young animals, but biased genotypic distributions with normal litter sizes and the absence of dead embryos support non-random fertilization rather than lethality after fertilization^8^. In cattle, there is usually only one calf per pregnancy and detecting embryo losses before approximately 28 days of gestation is difficult. Although data before day 28 are sparse, in dairy cows, fertilization failure appears to occur in 10–20% of AI, whereas approximately 35% of embryos are lost between days 8 and 60 of after AI, skewed to earlier times^20^. A similar study in pigs or mice would more easily distinguish between failure of fertilization and embryo lethality.

Insights into important processes related to gametic incompatibility can be gained from the overrepresentation analyses even though the results were not significant after FDR correction for multiple tests. The number of identified processes did not increase with the consideration of the overrepresentation analysis based on the KEGG pathways, i.e. the resulting pathways were subsets or on a par with mechanisms of the other analyses with significant results. Although the results from the overrepresentation analysis based on the KEGG pathways should be interpreted with caution, they can add additional information for the interpretation of the significant results from the other analyses in combination with the available literature for deciphering important gametic incompatibility mechanisms. No conclusions should be drawn solely from the non-statistically significant results of the overrepresentation analysis based on the KEGG pathways.

The pathway with the highest enrichment ratio was “Glycosphingolipid biosynthesis” (FDR = 0.24, Ratio = 5.37). Glycosphingolipids are important for cell membrane structure, the modulation of membrane protein function and external cell communication (host–pathogen interaction and cell–cell recognition)^21^. Genes associated with their biosynthesis are highly conserved in humans and mutated genes are associated with excessive apoptosis during embryo development or are correlated with diverse cancers later in life^21^. Another interesting term is “Endocytosis” (FDR = 0.24, Ratio = 1.81), which relates to functions similar to the aggresome and the autophagy processes, which were significant in the network analysis. After gametic fusion, there is an important release of cortical granules as compensatory endocytosis to maintain the cell surface and avoid polyspermy^22,23^. Without proper function of this mechanism, egg activation is compromised^22^.

More generally, the enriched pathways were related directly to immunologic functions or their dysfunctions linked to diseases. The two KEGG pathways with a highest enrichment ratio were “Graft-versus-host disease” (FDR = 0.26, Ratio = 2.92) and “Type I diabetes mellitus” (FDR = 0.26, Ratio = 2.66). These pathways shared similarities with mechanisms important for reproduction. The pathway “Graft-versus-host disease” shares functions with foetal acceptance by the dam. Seven of the eight genes (*BOLA*, *BOLA-DOA*, *BOLA-DMB*, *BOLA-DMA*, *BOLA-NC1*, *LOC512672*, *JSP.1*) are part of the major histocompatibility complex (MHC), a set of highly polymorphic genes present on BTA 23 coding for self- and non-self-recognition^24,25^. The last of the eight genes codes for interferon-gamma (IFN-γ), which plays a role in the foetal immuno-acceptance and male fertility^26,27^. Type I diabetes mellitus, which involves auto-immunity, also involves the same MHC and IFN-γ genes. Furthermore, five out of ten overrepresented KEGG pathways included a combination of the MHC genes above. Recent studies have concluded that MHC genes may mediate mate selection at both the individual and molecular levels in different ways (reviewed in Ref.^5^). In dairy cattle, the use of artificial insemination placed mating choices in human hands, removing the pre-copulatory sexual selection from the individual animals. However, mate selection also occurs post-copulation and pre-fertilization with the interaction of the male gamete with both the female reproductive tract and gamete. The most relevant example of the importance of gametic compatibility in mammals is the Izumo and Juno gene pair, coding for sperm surface protein and complementary egg receptor, respectively^4^. The two genes affect the sperm-egg fusion capacity, but not other aspects of fertilization, meaning that current sperm and oocyte quality evaluations done in the dairy industry would not detect anomalies. Discovering genes and mechanisms of gametic incompatibility would allow better prediction of fertility and mating outcomes. Moreover, further studies may allow for the identification of haplotypes with their specific compatibilities to others. This information could be later used as a tool for decision making in mate allocation and, in longer term, to select for individuals with higher general compatibility in the population.

The intent of the guilt-by-association analysis was to focus on the mechanisms of the mate selection within the reproductive tract. Because there are few genes associated with gametic incompatibility in the literature^4^, a thorough discussion of both candidate and prioritized genes follows. Most genes could be divided into three groups based on which fertilization mechanisms they influence: Gain of fertilization ability of the sperm (Group 1), Gametic interaction (Group 2), and Female immuno-acceptance (Group 3). The first mechanism relates to the sperm maturation and activation (Group 1) in the female reproductive tract and in proximity to the oocyte. Calcium signalling is important for sperm motility as it mediates the increased intracellular calcium necessary for capacitation, acrosome reaction, and hypermotility^28^. Both *CATSPERD* and *ATP2B4*, also called Plasma membrane calcium ATPase 4 (*PMCA4*), participate in this process and are associated with sperm fertilization success in mammals^28,29^. *SLC9B1* regulates intracellular pH, which is linked to the intracellular calcium concentration^30,31^. It is important to note that the different steps of sperm activation must be triggered at specific times in the female reproductive tract for the sperm cells to gain their fertilizing ability. For example, the acrosome reaction and hypermotility should be triggered only when the sperm reaches the zona pellucida (ZP), a protein layer protecting the oocyte, for fertilization^32^. In males, *PCSK4* protects against such premature acrosome reaction associated with lesser binding to the ZP^33^. Additionally, different *PRND* genotypes have been significantly associated with different acrosome reaction patterns, leading to differences in fertilization rates in rams^34^. The expression of *PRND* is also associated with the cryoresistance of sperm in sheep^34^.

After the proper activation and maturation of the spermatozoon, the interaction of the female and male gametes (Group 2) could reveal pair incompatibility. *CD9* is a well-known gene expressed on the oocyte membrane that defines the fusion ability of the gametes^35,36^, while *MFGE8* mediates sperm adhesion to the ZP^35^. Early embryo development and attachment is the next step where a gametic incompatibility may be expressed. By sequence similarity, *GNPDA1* is thought to play a role in calcium oscillations necessary for the egg activation^37^ and *UBE2Q1* is involved in female hormonal homeostasis and embryoid body formation^38^. In addition to its gamete interaction function, *MFGE8* is also involved in the regulation and maintenance of the endometrium to prepare for embryo attachment^39^. *MAEL* is mostly known for its link with spermatogenesis, but it is also associated with germ-cell differentiation within the embryo^40^. In females, *PCSK4* contributes to the development of follicles and may promote placentation^33^. The *MET* protein, also called hepatocyte growth factor receptor, has a PCSK-mediated activation in the male germ-line^33^ and is associated with abnormal mitosis in endometriosis, which may affect the receptivity of the endometrium to an embryo^41^.

Lastly, the immuno-acceptance of the allogenic cells (Group 3), i.e. male gamete and embryo, by the female reproductive tract is critical. Consistent with the overrepresentation analysis, *IFNG* was a prioritized candidate gene for gametic incompatibility. Levels of IFN-γ in the maternal serum can be used to predict the success of the early pregnancy^27,42^. *ANXA1* is an anti-inflammatory protein that regulates the secretion of steroids. Its knockout (KO) in female mice revealed a female-skewed sex ratio and larger litter size, but no functional alterations were observed in KO males^43^. Knockout of *ANXA1* changed the uterine inflammatory profile to promote early maternal–fetal interactions and implantation^43^.

The implications of other genes in gametic incompatibility were more complex to determine. *SMAD4* does not have a known major direct effect on fertility, but it takes part in the regulation of gametic and embryo development^44–46^. *MCM9* is associated with premature ovarian failure due to a deficit of the germ cell renewal^47^, and this deficiency results in genomic instability due to a reduction in the replication quality check, which promotes cancer in adults and negatively affects germ-line stem cells^47^. *DMC1* is a meiotic gene that has been associated with infertility in humans and mice^48,49^ but to our knowledge, the mechanism behind this infertility has yet to be linked to any system dysfunctions. Allelic incompatibility, within or between loci, would be a promising hypothesis to describe this unexplained infertility. It is important to note that the choice of keywords used in the analysis is limited to current knowledge of the investigated phenotype. Therefore, the results of the analysis can only pinpoint genes that have already been associated with or are suspected to be associated with mechanisms of gametic incompatibility. As gametic incompatibility is a novel phenotype in livestock, and even in humans, little is known about its genetic background and only a few candidate genes have been identified^4^. With further investigation into the mechanisms, more keywords will be identified, and the candidate gene list will become more comprehensive.

## Conclusion

Gametic incompatibility in dairy cattle partially explains unequal genotype ratios in the offspring generation. Based on recognition and immunological functions within the reproductive tract, fertilization is not always a random event as commonly assumed. An animal’s probability of fertilization is therefore not only predicted by its innate fertility, but also by its mate compatibility. Currently, the industry does not account for gametic incompatibility, reducing the accuracy of prediction of fertility at the individual and gametic levels. With further investigation, the identification of compatible haplotype pairs and prediction of mating success could become possible, improving mate allocation in dairy cattle.

## Materials and methods

### Dataset

To study TRD effects linked to gametic incompatibility, a dataset from a study that identified TRD regions associated with reproduction defects was used^50^. This dataset consisted of 436,651 genotyped Canadian Holstein cattle (5976 sires, 132,282 dams and 283,817 offspring) provided by Lactanet (Guelph, Canada), which composed of 283,817 dam-sire-offspring trios. Both parents could be part of different trios with an average of 57.07 and 2.57 trios per sire and dam, respectively. Only the first offspring for a specific mating was considered. Only animals with offspring genotyped within 90 days of birth were selected to avoid a bias associated with the phenotypic preselection of the individuals to be genotyped within a family. The animals were genotyped with different single nucleotide polymorphism (SNP) genotyping arrays and low-density genotypes were imputed with Fimpute^51^ to 47,910 SNPs. The genotypes and imputed genotypes were based on the genome assembly UMD3.1.

### Transmission ratio distortion model

Under the hypothesis that some allelic TRD signals could be caused by gametic incompatibility, regions previously identified with direct TRD^50^ could be analysed with an alternative model considering the interaction between gametes in the offspring generation. Thus, the inheritance of alleles from parent to offspring could be parametrized including a direct TRD effect ($\alpha_{ij}$) and an interaction between offspring alleles ($\beta_{ij}$). In this case, the probability of an offspring ($P_{off}$) in a locus with $n$ alleles must be parameterized for each specific mating. Assuming a locus with four alleles and a mating of heterozygous parents with different alleles ($A_{1} A_{2}$ × $A_{3} A_{4}$),

$$P_{off} \left( {A_{1} A_{3} } \right) = \left[ {\left( {0.5 + \alpha_{12} } \right) \times \left( {0.5 + \alpha_{34} } \right)} \right] \times \left( {1 + \beta_{13} } \right) \times \left( {1 - \left( {\beta_{14} /3} \right)} \right) \times \left( {1 - \left( {\beta_{23} /3} \right)} \right) \times \left( {1 - \left( {\beta_{24} /3} \right)} \right)$$

$$P_{off} \left( {A_{1} A_{4} } \right) = \left[ {\left( {0.5 + \alpha_{12} } \right) \times \left( {0.5 - \alpha_{34} } \right)} \right] \times \left( {1 - \left( {\beta_{13} /3} \right)} \right) \times \left( {1 + \beta_{14} } \right) \times \left( {1 - \left( {\beta_{23} /3} \right)} \right) \times \left( {1 - \left( {\beta_{24} /3} \right)} \right)$$

$$P_{off} \left( {A_{2} A_{3} } \right) = \left[ {\left( {0.5 - \alpha_{12} } \right) \times \left( {0.5 + \alpha_{34} } \right)} \right] \times \left( {1 - \left( {\beta_{13} /3} \right)} \right) \times \left( {1 - \left( {\beta_{14} /3} \right)} \right) \times \left( {1 + \beta_{23} } \right) \times \left( {1 - \left( {\beta_{24} /3} \right)} \right)$$

$$P_{off} \left( {A_{2} A_{4} } \right) = \left[ {\left( {0.5 - \alpha_{12} } \right) \times \left( {0.5 - \alpha_{34} } \right)} \right] \times \left( {1 - \left( {\beta_{13} /3} \right)} \right) \times \left( {1 - \left( {\beta_{14} /3} \right)} \right) \times \left( {1 - \left( {\beta_{23} /3} \right)} \right) \times \left( {1 + \beta_{24} } \right)$$

where $\alpha_{12}$ and $\alpha_{34}$ were the heterozygous pairwise combinations of direct TRD effects of the implicated parents; $\beta_{ij}$ was the interaction of alleles $i$ and $j$ in the offspring generation. Note that 1, 2, 3 and 4 were the alleles implicated in this mating.

The direct effects described the probability of transmission of one allele at the expense of the opposite allele in the heterozygous pairwise combination. Flat priors were assumed within a parametric space ranging from − 0.5 to 0.5, based on the principles of Mendelian inheritance. The probability of transmission of one specific allele ranged from 0 ($\alpha_{ij}$ =  − 0.5) to 1 ($\alpha_{ij}$ = 0.5), where 0.5 ($\alpha_{ij}$ = 0) corresponds to no TRD.

The TRD parameters of each specific alleles’ interaction in the offspring genotype were included to model the incompatibility of the offspring genotype. For gametic incompatibility, certain alleles’ interactions should be underrepresented and display a genotype interaction TRD pattern.

The gametic interaction TRD parameter indicated the probability of an offspring to be produced and viable. This parameter was assumed to range from -1 (decreased probability) to + 1 (increased probability). A magnitude of 0 indicated no gametic interaction TRD. Note that when one offspring was over- or under-represented, the change in the frequency was compensated by one or several other offspring genotypes of the same mating.

For the gametic interaction TRD parameters, flat priors were also assumed. The probabilities of offspring genotypes must be adapted for each specific mating, but could be generalized as:

$${\text{P}}_{{{\text{off}}}} ~\left( {{\text{A}}_{{{\text{s}}_{1} }} {\text{A}}_{{{\text{d}}_{1} }} } \right) = ~\left[ {\left( {0.5 + \upalpha _{{{\text{s}}_{1} ,{\text{s}}_{2} }} } \right) \times \left( {0.5 + \upalpha _{{{\text{d}}_{1} ,{\text{d}}_{2} }} } \right)} \right] \times \left( {1 + \upbeta _{{{\text{s}}_{1} ,{\text{d}}_{1} }} } \right) \times \left( {1 - \left( {\upbeta _{{{\text{s}}_{1} ,{\text{d}}_{2} }} /3} \right)} \right) \times \left( {1 - \left( {\upbeta _{{{\text{s}}_{2} ,{\text{d}}_{1} }} /3} \right)} \right) \times \left( {1 - \left( {\upbeta _{{{\text{s}}_{2} ,{\text{d}}_{2} }} /3} \right)} \right)$$

$${\text{P}}_{{{\text{off}}}} ~\left( {{\text{A}}_{{{\text{s}}_{1} }} {\text{A}}_{{{\text{d}}_{2} }} } \right) = ~\left[ {\left( {0.5 + \upalpha _{{{\text{s}}_{1} ,{\text{s}}_{2} }} } \right) \times \left( {0.5 - \upalpha _{{{\text{d}}_{1} ,{\text{d}}_{2} }} } \right)} \right] \times \left( {1 - (\upbeta _{{{\text{s}}_{1} ,{\text{d}}_{1} }} /3)} \right) \times \left( {1 + \upbeta _{{{\text{s}}_{1} ,{\text{d}}_{2} }} } \right) \times \left( {1 - \left( {\upbeta _{{{\text{s}}_{2} ,{\text{d}}_{1} }} /3} \right)} \right) \times \left( {1 - \left( {\upbeta _{{{\text{s}}_{2} ,{\text{d}}_{2} }} /3} \right)} \right)$$

$${\text{P}}_{{{\text{off}}}} ~\left( {{\text{A}}_{{{\text{s}}_{2} }} {\text{A}}_{{{\text{d}}_{1} }} } \right) = ~\left[ {\left( {0.5 - \upalpha _{{{\text{s}}_{1} ,{\text{s}}_{2} }} } \right) \times \left( {0.5 + \upalpha _{{{\text{d}}_{1} ,{\text{d}}_{2} }} } \right)} \right] \times \left( {1 - (\upbeta _{{{\text{s}}_{1} ,{\text{d}}_{1} }} /3)} \right) \times \left( {1 - \left( {\beta _{{{\text{s}}_{1} ,{\text{d}}_{2} }} /3} \right)} \right) \times \left( {1 + \upbeta _{{{\text{s}}_{2} ,{\text{d}}_{1} }} } \right) \times \left( {1 - \left( {\upbeta _{{{\text{s}}_{2} ,{\text{d}}_{2} }} /3} \right)} \right)$$

$${\text{P}}_{{{\text{off}}}} ~\left( {{\text{A}}_{{{\text{s}}_{2} }} {\text{A}}_{{{\text{d}}_{2} }} } \right) = ~\left[ {\left( {0.5 - \upalpha _{{{\text{s}}_{1} ,{\text{s}}_{2} }} } \right) \times \left( {0.5 - \upalpha _{{{\text{d}}_{1} ,{\text{d}}_{2} }} } \right)} \right] \times \left( {1 - (\upbeta _{{{\text{s}}_{1} ,{\text{d}}_{1} }} /3)} \right) \times \left( {1 - \left( {\upbeta _{{{\text{s}}_{1} ,{\text{d}}_{2} }} /3} \right)} \right) \times \left( {1 - \left( {\upbeta _{{{\text{s}}_{2} ,{\text{d}}_{1} }} /3} \right)} \right) \times \left( {1 + \upbeta _{{{\text{s}}_{2} ,{\text{d}}_{2} }} } \right)$$

where $\alpha_{ij}$ was the heterozygous pairwise combination of direct TRD effect; ${\upbeta }_{{{\text{ij}}}}$ was the gametic interaction TRD parameter; ${\text{s}}_{1}$ and ${\text{s}}_{2}$ were the alleles from the sire; and ${\text{d}}_{1}$ and ${\text{d}}_{2}$ were the alleles from the dam.

For a specific mating, a maximum of 4 gametic interaction TRD parameters were involved. When all TRD effects were null, the probability of each allelic combination for a genotype was 0.25 and summed to 1 for the 4 possible combinations of genotypes. It is important to remember that the same offspring’s genotype may be generated in more than one of these 4 possible combinations and must be added to obtain the probability of each offspring’s genotype. Under a Bayesian implementation, the conditional posterior probabilities of the TRD parameters were defined as:

$$p\left( {\alpha_{12} ,\alpha_{13} , \ldots \alpha_{{\left( {n - 1} \right)n}} ,\beta_{11} , \beta_{12} , \ldots \beta_{nn} | y} \right) \propto p\left( {y | \alpha_{12} ,\alpha_{13} , \ldots \alpha_{{\left( {n - 1} \right)n}} ,\beta_{11} , \beta_{12} , \ldots \beta_{nn} } \right)p\left( {\alpha_{12} } \right)p\left( {\alpha_{13} } \right) \ldots p\left( {\alpha_{{\left( {n - 1} \right)n}} } \right)p\left( {\beta_{11} } \right)p\left( {\beta_{12} } \right) \ldots p\left( {\beta_{nn} } \right)$$

where ${\varvec{y}}$ was the vector of genotypes of the offspring generation.

As an exhaustive search for gametic interaction TRD is computationally intractable, the estimation was based on 602 regions previously identified with significant direct TRD effect^50^. The analysis was performed within a Bayesian framework with the metropolis-Hastings^52^ sampling technique using an adapted version of TRDscan v.1.0 software^53^. Each parameter was sampled separately, assuming the other parameters to be known a priori. A unique Monte Carlo Markov chain of 110,000 iterations was used, with the first 10,000 discarded as burn-in. The statistical significance of TRD was evaluated using a Bayes factor (BF)^54^. A threshold of BF ≥ 100 was used to determine decisive evidence for TRD.

### Functional analysis

The regions were filtered based on the magnitude of the gametic interaction term. This value is specific to the haplotype combination within each region. Therefore, multiple allelic combinations within one region could be detected with significant gametic interaction TRD. Only regions with at least one combination with a magnitude less than − 0.5 or greater than 0.5 were retained.

As the TRD analysis was performed with genotypes based on the previous genome assembly, UMD3.1, the TRD regions were remapped to the current genome assembly, ARS-UCD1.2, using the NCBI remap tool (www.ncbi.nlm.nih.gov/genome/tools/remap) before performing the functional analyses. Regions with large insertions or low coverage after remapping were removed from the list. To ensure proper conversion of the regions, a comparison of the extracted annotations from the two assemblies was performed using the GALLO package^55^. The two gene extractions overlapped by 85% of the annotations, and the remaining 15% were composed of repetitive elements, such as pseudogenes, or annotations without assigned gene symbols.

The positional genes were then extracted based on the chromosomal positions of the remapped regions using the GALLO package^55^. An additional 50,000 bp were added up and downstream to each region. The extracted list was used to perform a network analysis with the NetworkAnalyst software^14^. The study was performed with the Ensembl gene IDs, using a protein–protein interactions (PPI) analysis. This approach used predefined networks from the STRING interactome database^56^ to build a network from the input gene list and other proteins. This PPI analysis created a broad view of the biological mechanisms linked to the investigated phenotype, in this case, gametic incompatibility.

A minimum interaction network that kept the essential proteins needed to maintain the network connections and minimize the use of enriched proteins was created. A biological processes analysis was performed to identify significantly overrepresented biological pathways within the gene list through the NetworkAnalyst software^14^ based on GO terms and the KEGG pathways.

To further the investigation of gametic incompatibility, a “guilt-by-association”-based prioritization analysis was performed on the positional gene list using GUILDify 2.0^57^ and ToppGene^58^. First, GUILDify was used to create a trained list of the positional genes associated with specific keywords relating to the phenotype, i.e. “fertilization”, “gamete interaction”, “single fertilization” and “sperm-egg recognition”. The definition and potential implication of the genes in gametic incompatibility were investigated with the GeneCards database^59^ to confirm their relevance within the trained list. This trained list was then fitted into ToppGene against the full list of positional genes, for an annotation-based prioritization analysis. This is a multivariate approach that scores the functional profile similarity of each positional gene with the trained list profile, based on Gene Ontology terms for molecular function (MF), biological process (BP), and cellular component (CC); human and mouse phenotypes; metabolic pathways; Pubmed publications; Coexpression atlas; ToppCell atlas; and diseases. Intermediate p-values for each of the above functional terms and pathways, were obtained and combined into an overall p-value for each gene. This information was calculated by comparing the gene list with a random sample of 5000 genes from the whole genome for each annotation information. Then, the overall p-value was corrected for multiple testing using an FDR of 5%. Significantly prioritized genes shared a similar functional profile with the genes from the trained list. As the genes from the trained list were known to be associated with the phenotype, the prioritized genes are likely to also be associated with gametic incompatibility under the principle of guilt-by-association.

Additionally, an over-representation analysis (ORA) was performed to determine if any biological pathways were over-represented in the gene list. This was done by uploading the list of genes identified by their Ensembl gene ID to the WebGestalt software^60^. The significance of each pathway is defined by the difference between the number of genes observed and the expected number of genes that would have been obtained from a random set of genes of the same size given the whole genome annotation. The ratio of over-representation was also obtained and reported.

## Supplementary Information

Supplementary Figure S1.

Supplementary Table S1.

Supplementary Table S2.

Supplementary Table S3.

## Data Availability

The genotypic data analysed for the current study were obtained from Lactanet (Guelph, Canada). This dataset was used under agreement, and therefore, not publicly available. However, data can be made available from F.M. on reasonable request and with permission of Lactanet.
