# Supplementary material for: Unravelling the genetics of non-random fertilization associated with gametic incompatibility
Source: Sci Rep. 2022 Dec 24;12:22314. doi: 10.1038/s41598-022-26910-8 (PMC9789956; doi:10.1038/s41598-022-26910-8)

Figure S1. Protein-by-Protein minimum interaction networks based on genes positioned in transmission ratio distortion genomic regions associated with strong gametic interactions.


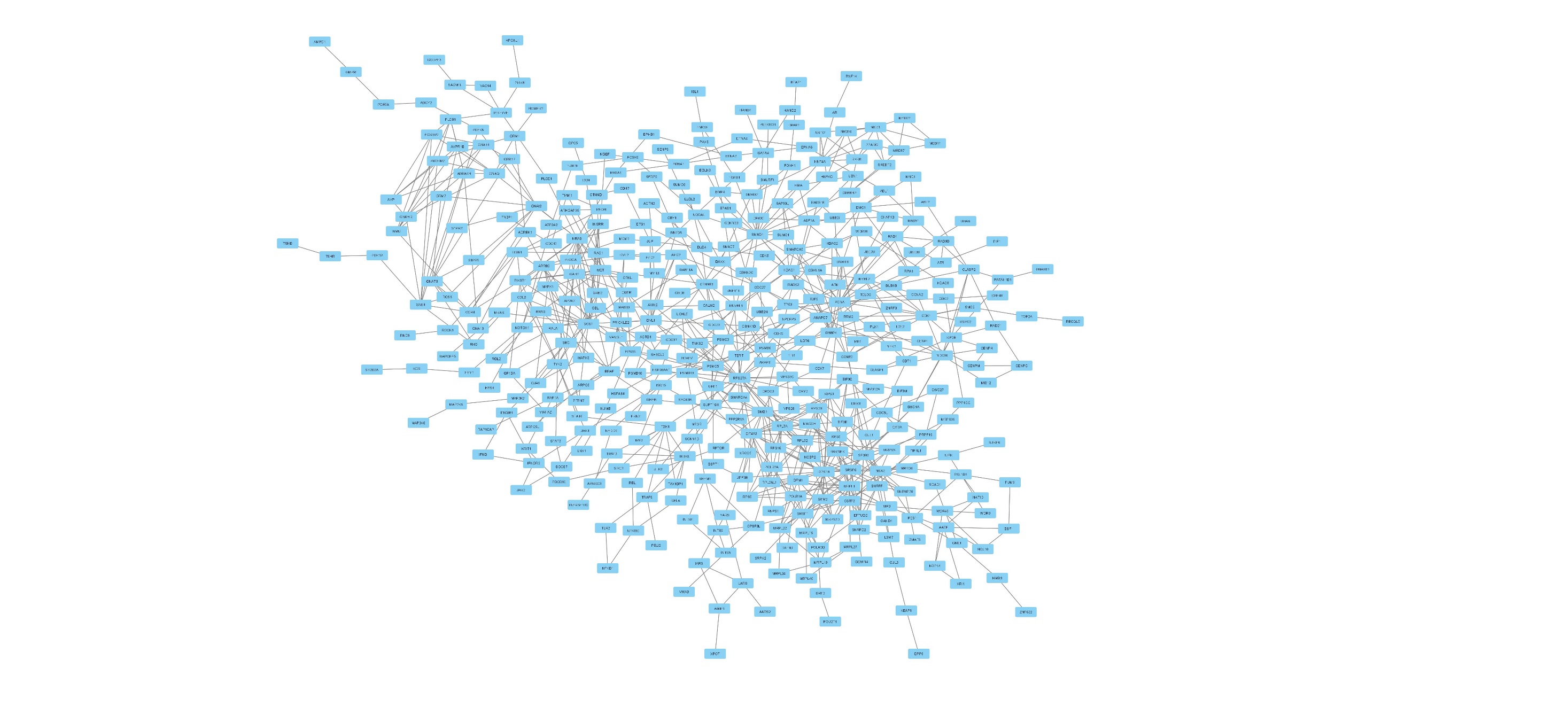
Subnetwork 1:

Subnetwork 2:


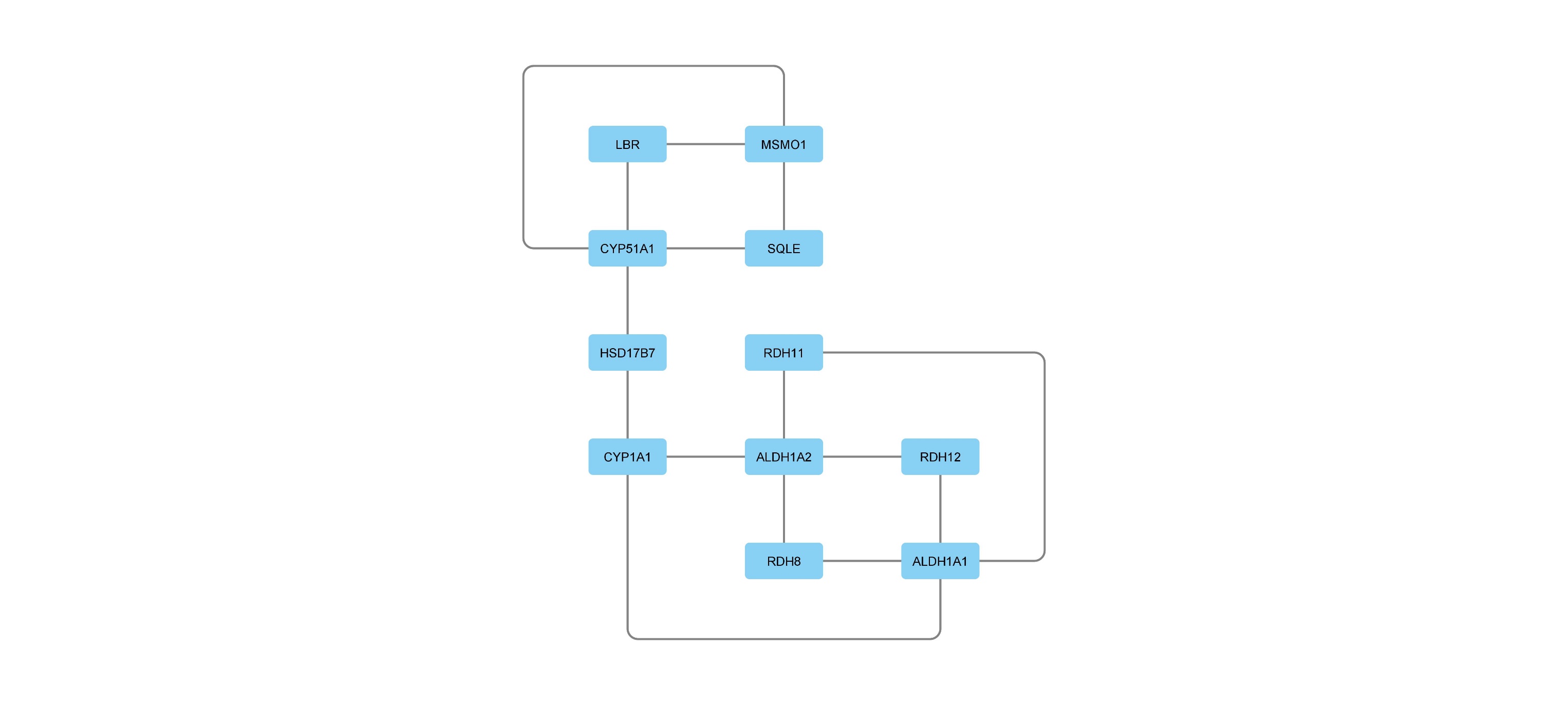


Subnetwork 3:


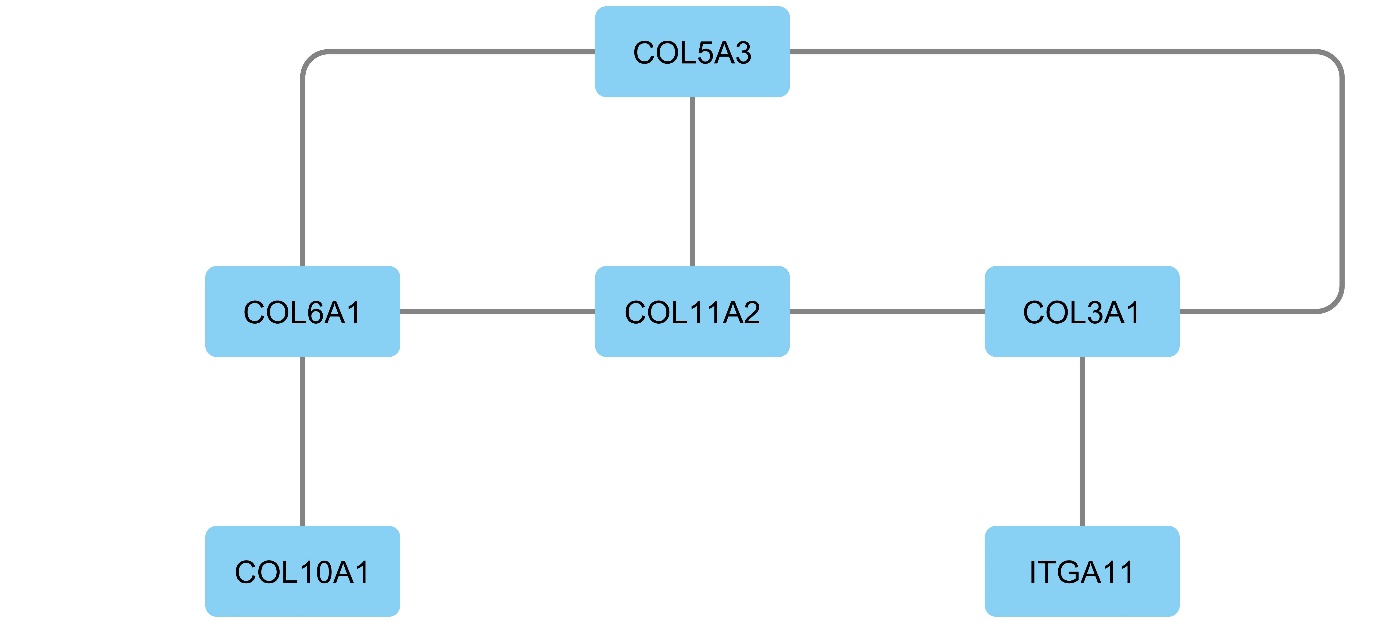


Subnetwork 4:


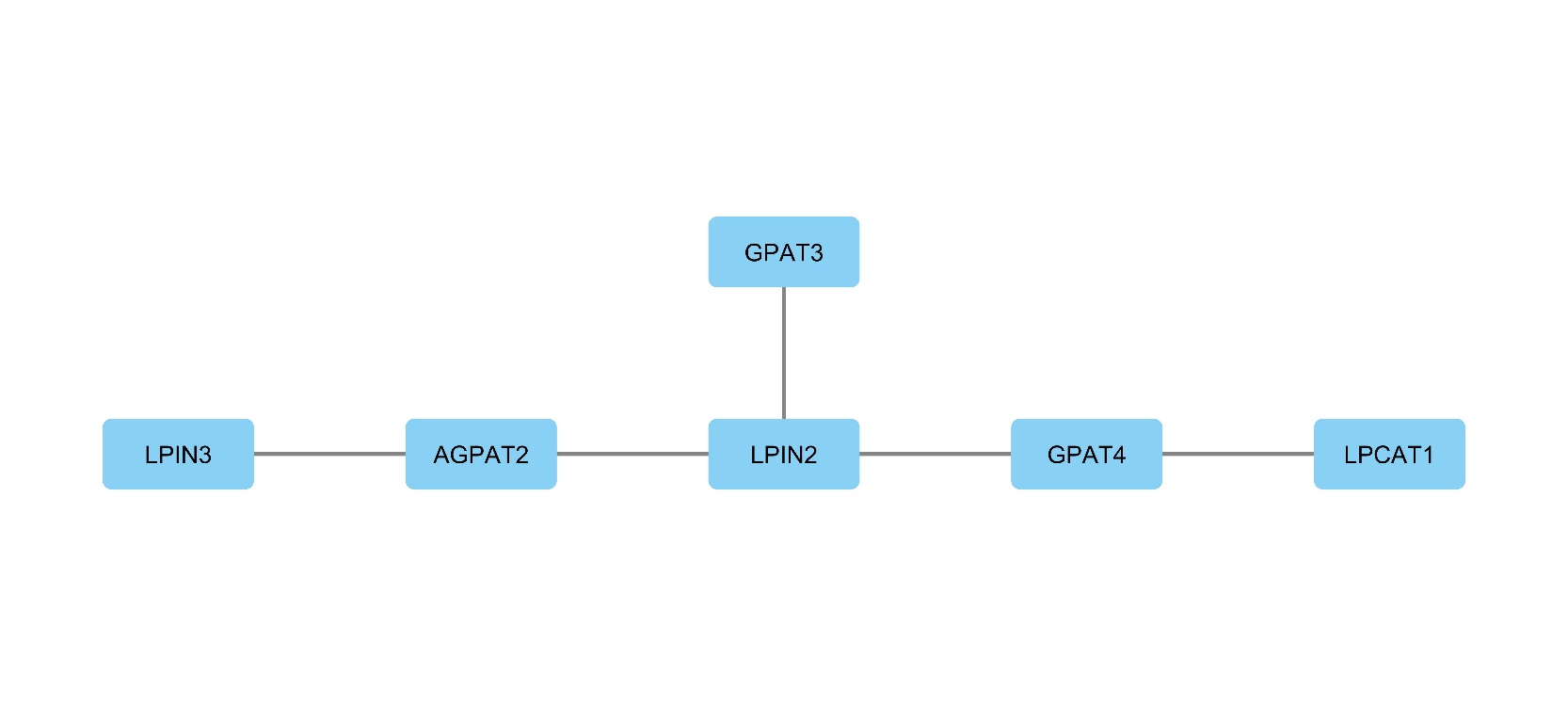


Subnetwork 5:


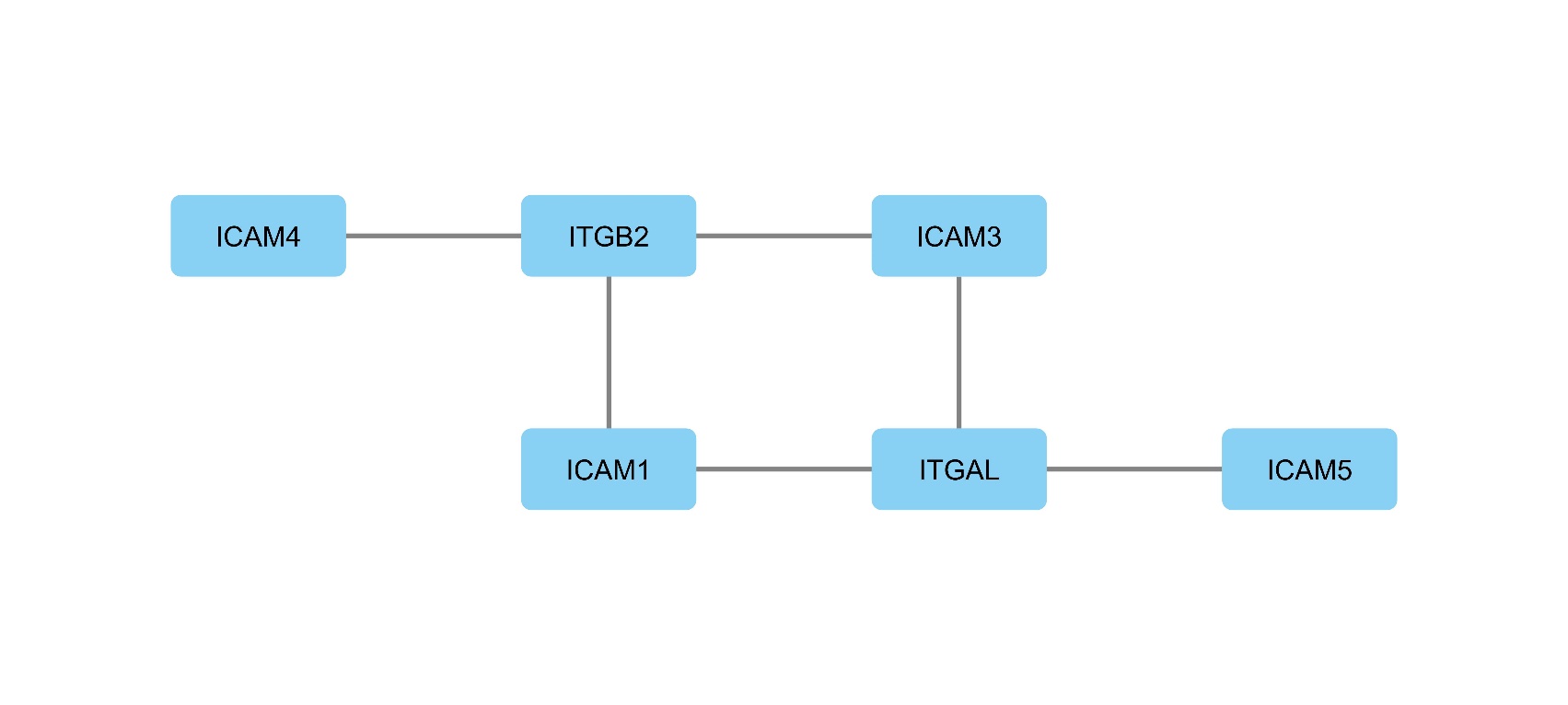


Subnetwork 6:


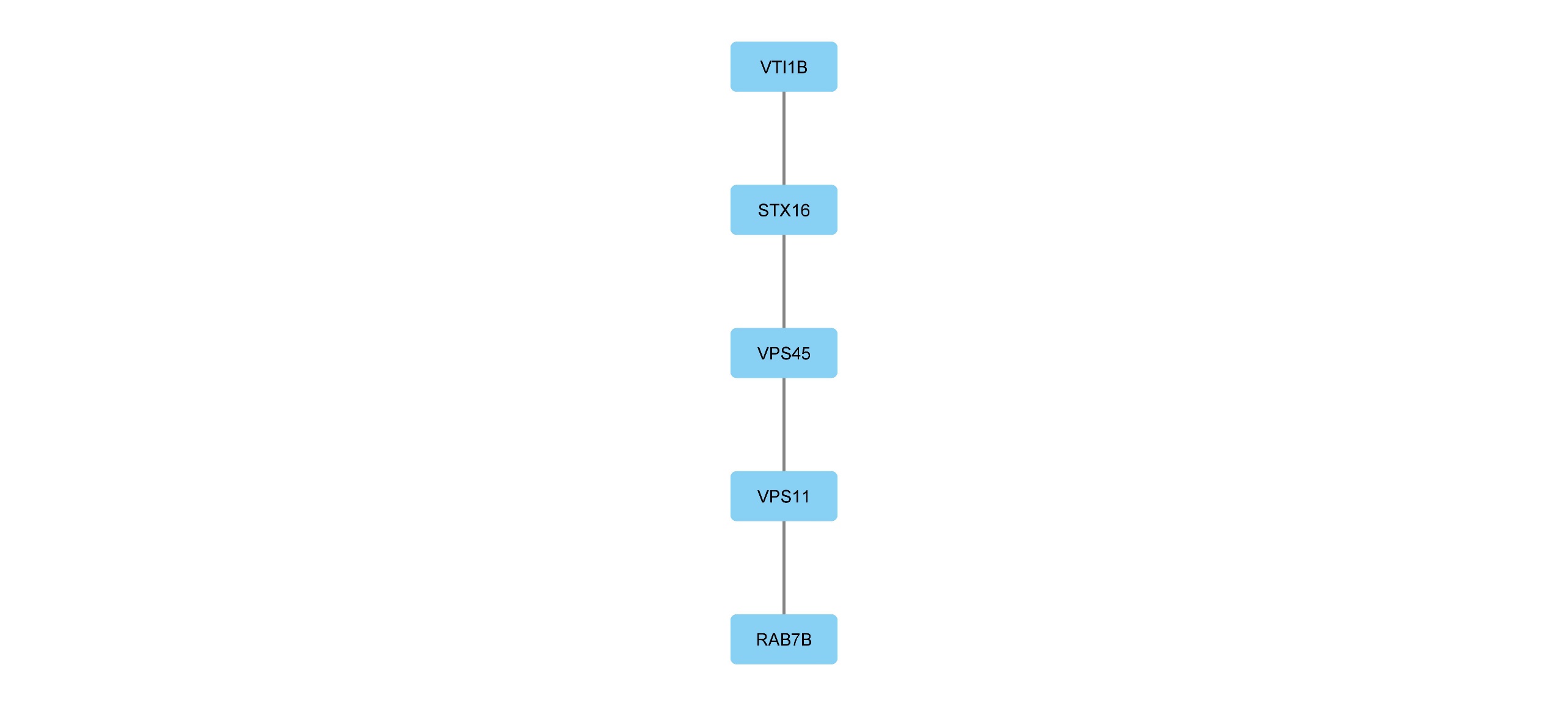


Subnetwork 7:


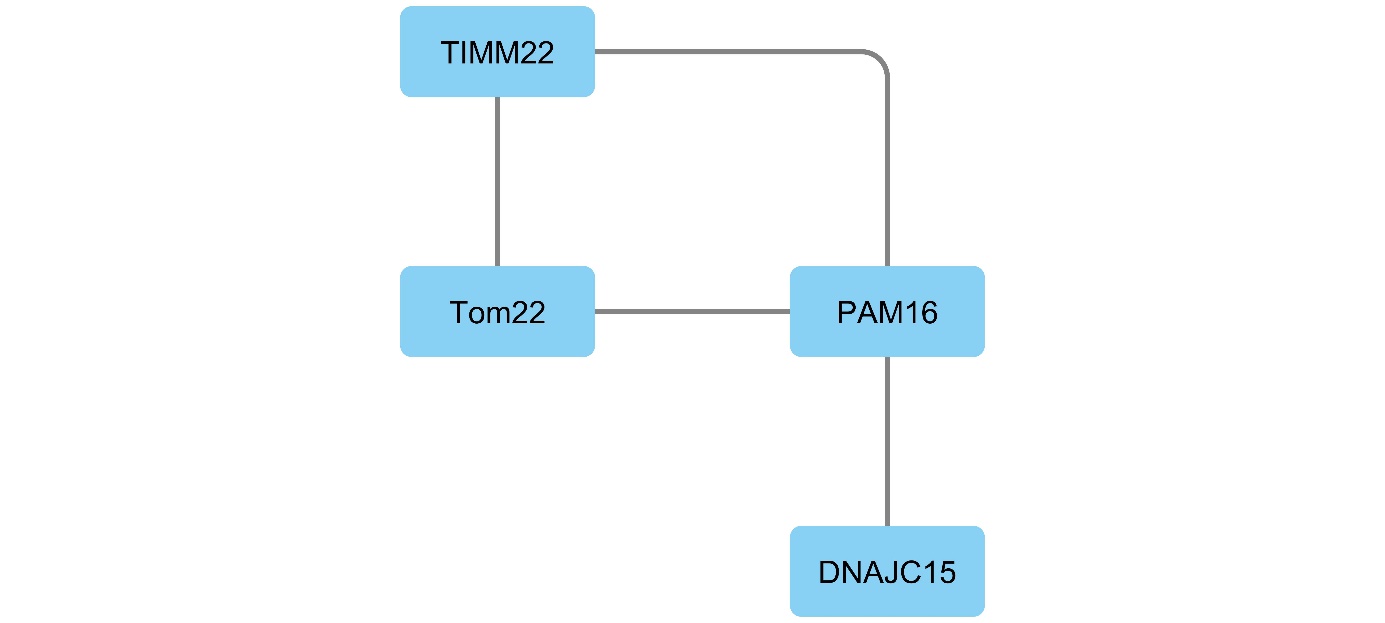


Subnetwork 8:


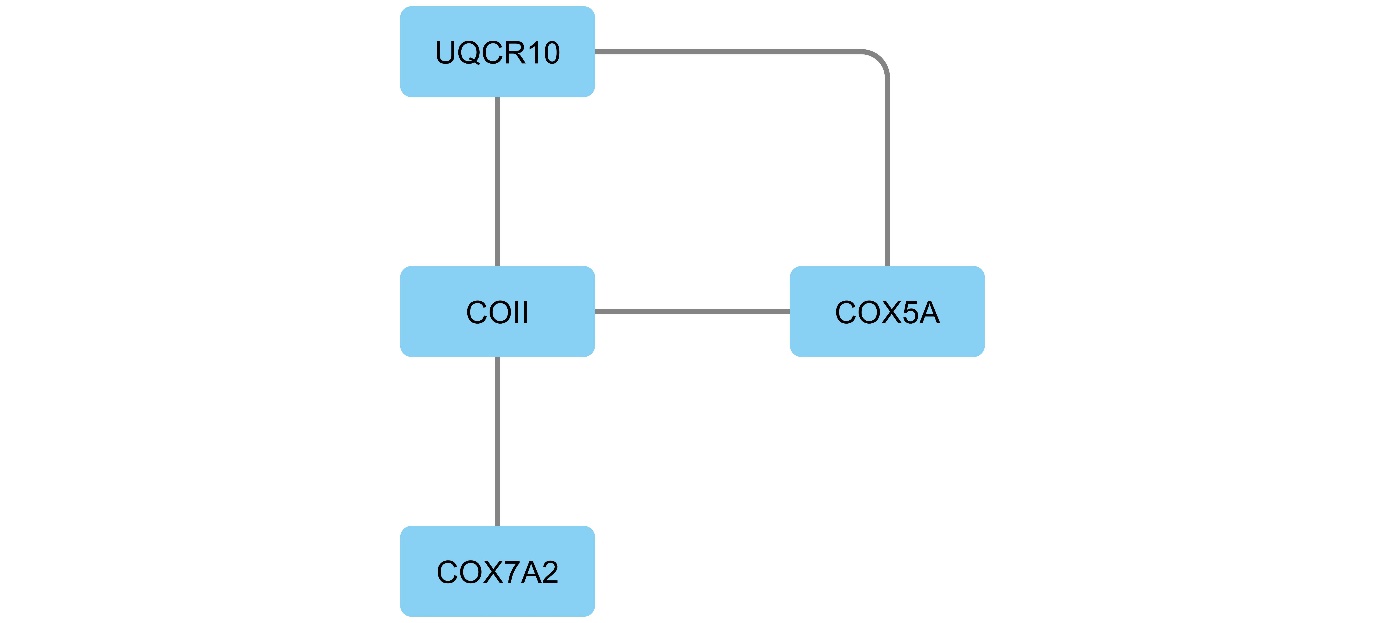


Subnetwork 9:


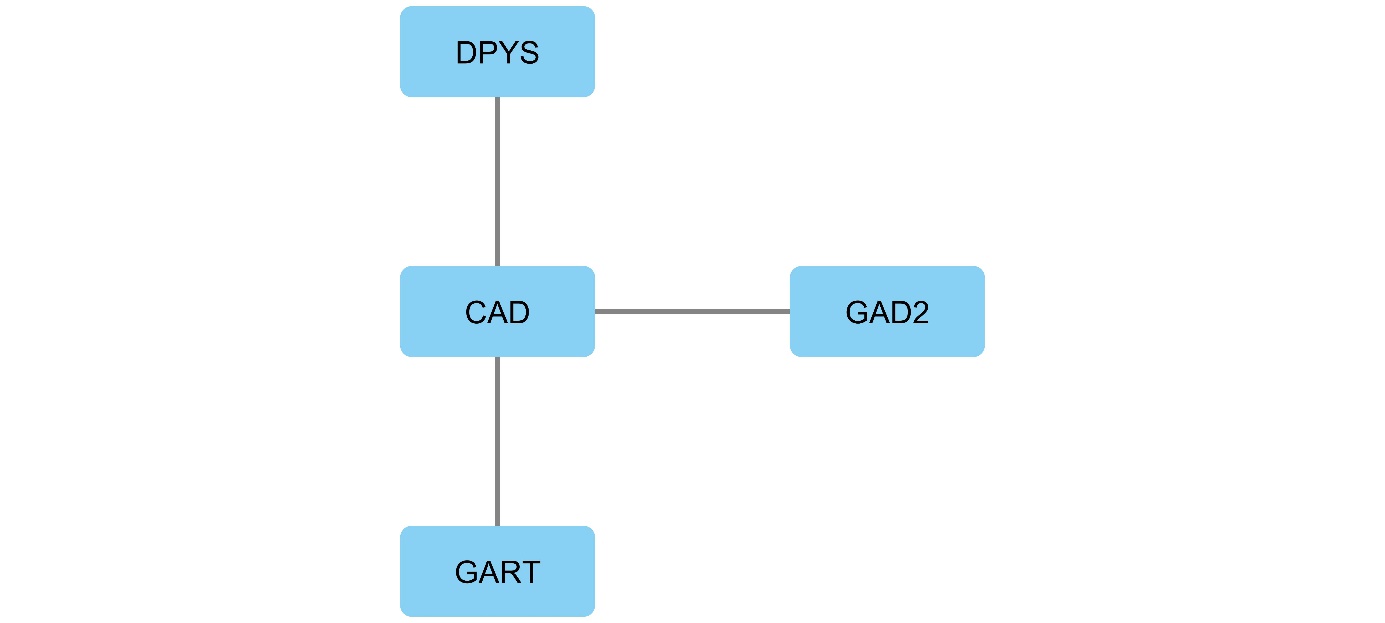


Subnetwork 10:


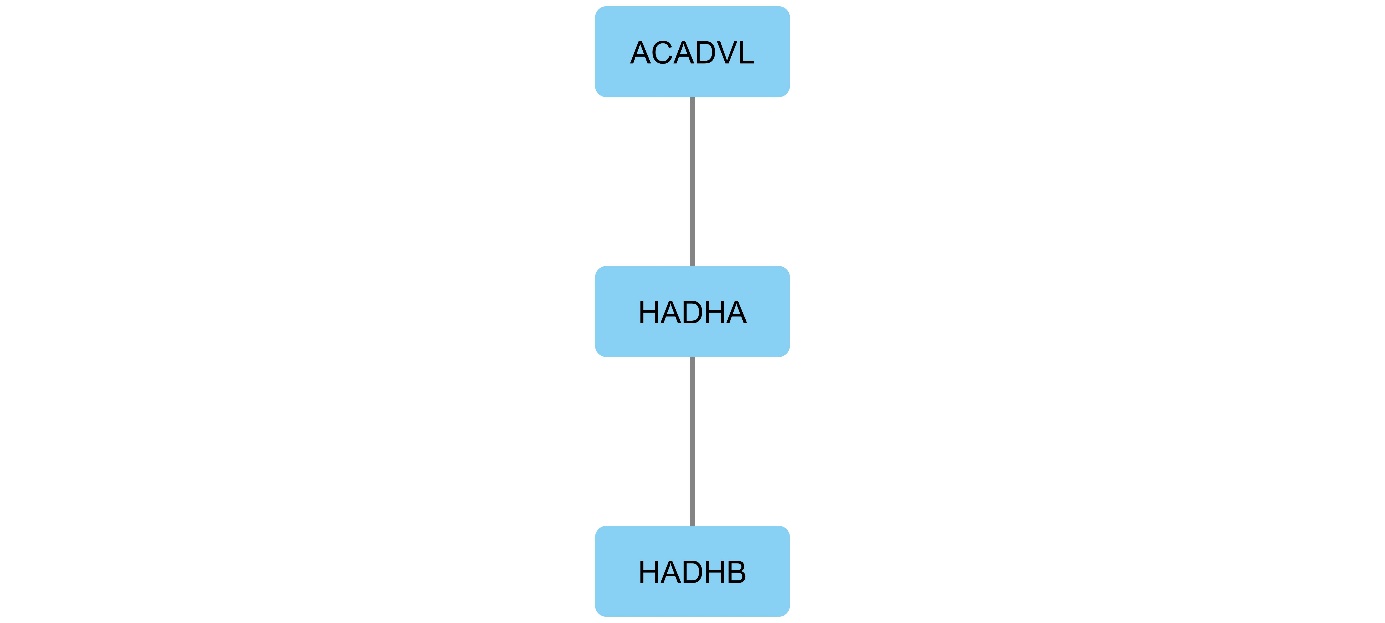


Subnetwork 11:


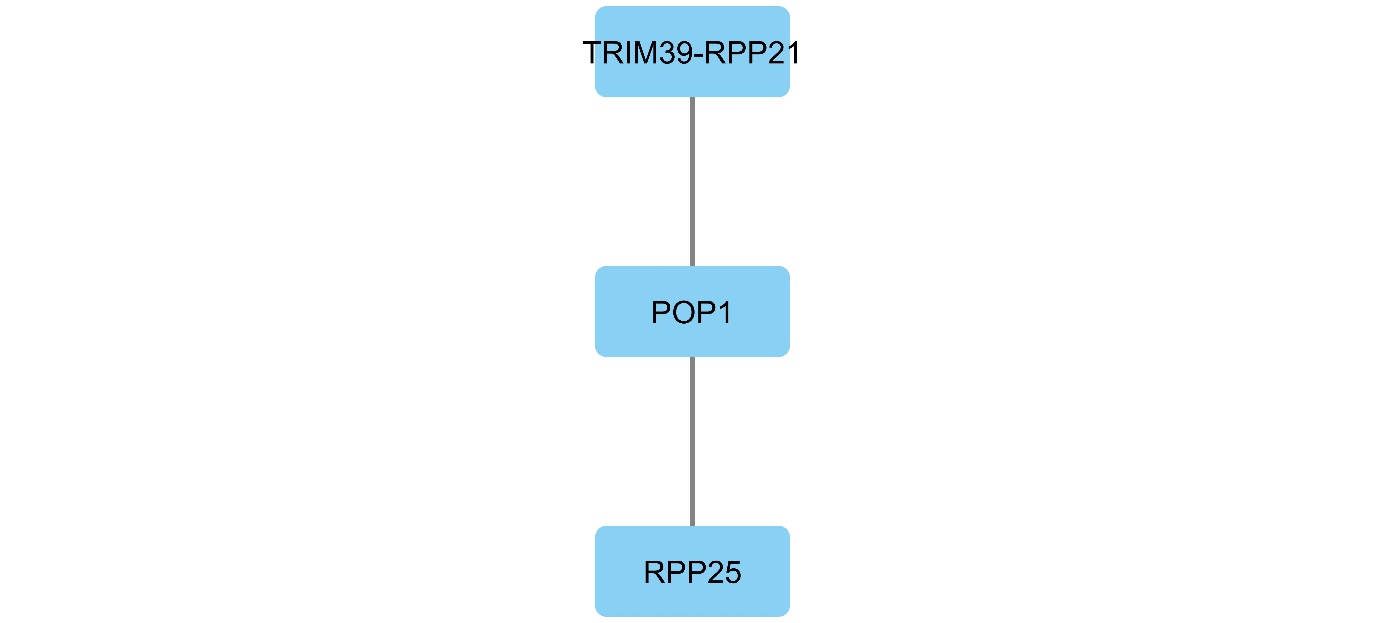


Subnetwork 12:


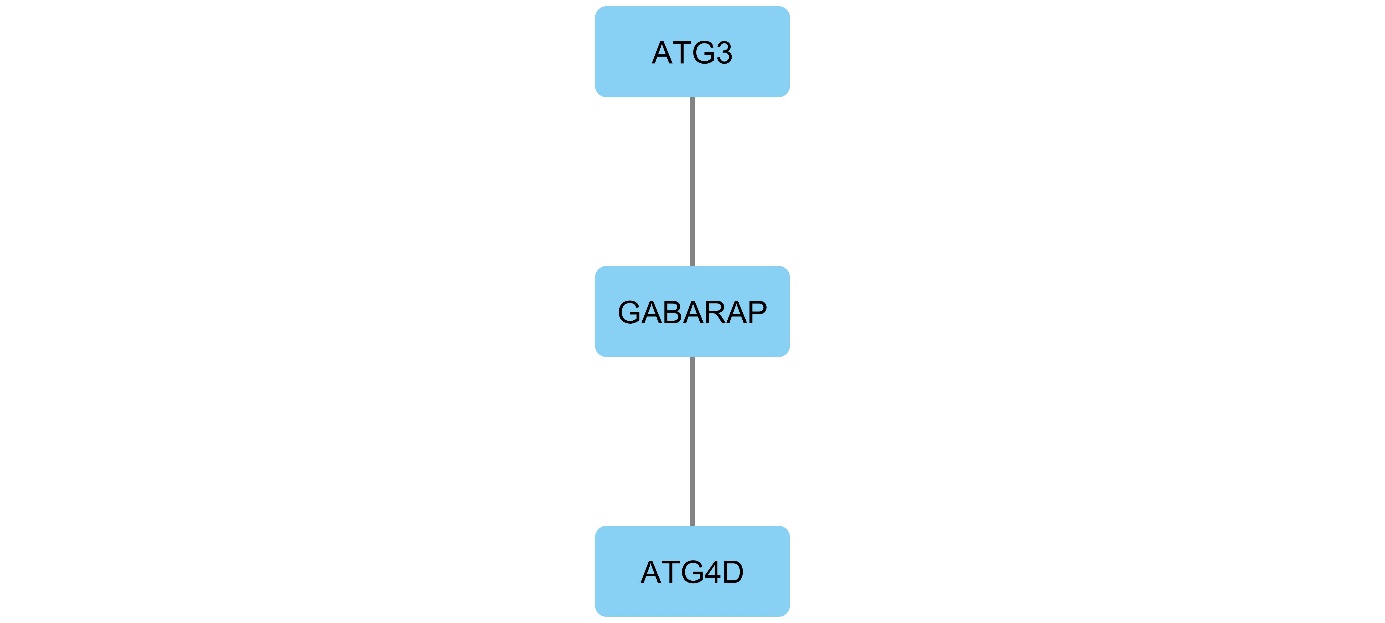


Subnetwork 13:


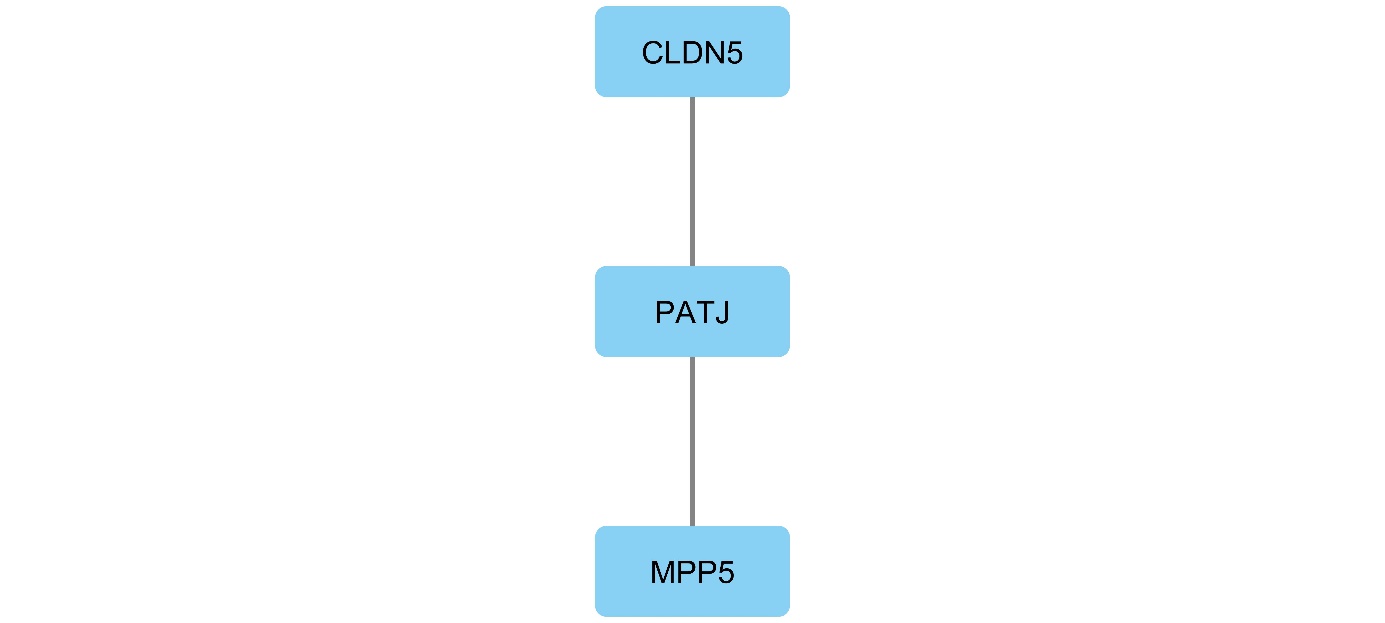

Supplement: Supplementary file 1 — Supplementary Figure S1. [file 41598_2022_26910_MOESM1_ESM.docx]
